# Supplementary material for: MetaRibo-Seq measures translation in microbiomes
Source: Nat Commun. 2020 Jun 29;11:3268. doi: 10.1038/s41467-020-17081-z (PMC7324362; doi:10.1038/s41467-020-17081-z)
Supplement: Supplementary file 10 — Supplementary Data 7 [file 41467_2020_17081_MOESM10_ESM.zip › File2/Confidence_High_Taxonomy/177836_out.krona.html]

Javascript must be enabled to view this page.

members
magnitude
magnitudeUnassigned
count
unassigned
taxon
rank

177836\_out

76

2
superkingdom
76

976
phylum
76

76
class
200643

76
order
171549

family
815
76

genus
816
76

species
817

SRS011084\_contig\_number\_23366SRS012902\_contig\_number\_69SRS013158\_contig\_number\_1429SRS013215\_contig\_number\_238SRS013476\_contig\_number\_31800SRS014613\_contig\_number\_10955SRS014736\_contig\_number\_7038SRS015190\_contig\_number\_14821SRS015264\_contig\_number\_19437SRS015578\_contig\_number\_422SRS015890\_contig\_number\_22295SRS015960\_contig\_number\_17269SRS016267\_contig\_number\_9237SRS017433\_contig\_number\_9741SRS017521\_contig\_number\_49736SRS017622\_contig\_number\_140SRS018313\_contig\_number\_10086SRS018541\_contig\_number\_889SRS018888\_contig\_number\_788SRS018984\_contig\_number\_10302SRS019267\_contig\_number\_8575SRS019601\_contig\_number\_25SRS019787\_contig\_number\_2354SRS021484\_contig\_number\_contig-100\_234.159228SRS022524\_contig\_number\_13425SRS023829\_contig\_number\_54SRS023971\_contig\_number\_1740SRS024132\_contig\_number\_37343SRS024549\_contig\_number\_4173SRS043411\_contig\_number\_2446SRS046502\_contig\_number\_11854SRS046712\_contig\_number\_323SRS048981\_contig\_number\_128SRS049402\_contig\_number\_13551SRS050299\_contig\_number\_13893SRS051610\_contig\_number\_1516SRS055966\_contig\_number\_1878SRS056259\_contig\_number\_16367SRS058723\_contig\_number\_3568SRS063040\_contig\_number\_31392SRS063127\_contig\_number\_1820SRS063190\_contig\_number\_1058SRS063985\_contig\_number\_12449SRS064276\_contig\_number\_35857SRS074964\_contig\_number\_4202SRS075021\_contig\_number\_6131SRS075398\_contig\_number\_331SRS075773\_contig\_number\_8735SRS076804\_contig\_number\_contig-100\_668.115705SRS077086\_contig\_number\_3852SRS077194\_contig\_number\_805SRS077335\_contig\_number\_648SRS078419\_contig\_number\_28445SRS098655\_contig\_number\_173SRS098717\_contig\_number\_23840SRS104036\_contig\_number\_3658SRS104311\_contig\_number\_28765SRS104485\_contig\_number\_564SRS104636\_contig\_number\_539SRS1054928\_contig\_number\_846SRS140492\_contig\_number\_33554SRS140513\_contig\_number\_1964SRS142599\_contig\_number\_30175SRS143342\_contig\_number\_9445SRS144362\_contig\_number\_9633SRS144506\_contig\_number\_398SRS147139\_contig\_number\_14049SRS147272\_contig\_number\_110SRS147377\_contig\_number\_3336SRS147557\_contig\_number\_26196SRS147653\_contig\_number\_2679SRS148159\_contig\_number\_259SRS893279\_contig\_number\_169SRS971275\_contig\_number\_2863
74


SRS024388\_contig\_number\_119
1
species
246787

1

SRS053573\_contig\_number\_1005
28116
species
